# Synthesis and Characterization of Novel Perfluoro Aromatic Side Chain Sulfonated PSU Ionomers

**DOI:** 10.1021/acspolymersau.4c00059

**Published:** 2024-10-14

**Authors:** Philipp Martschin, Vladimir Atanasov, Simon Thiele, Jochen Kerres

**Affiliations:** † Forschungszentrum Jülich GmbH, Helmholtz Institute Erlangen-Nürnberg for Renewable Energy (IET-2), Cauerstraße 1, 91058 Erlangen, Germany; ‡ Department of Chemical and Biological Engineering, Friedrich Alexander Universität Erlangen-Nürnberg, Egerlandstraße 3, 91058 Erlangen, Germany; § Institute of Chemical Process Engineering, University of Stuttgart, Böblinger Straße 78, 70199 Stuttgart, Germany; ∥ Chemical Resource Benefication Faculty of Natural Sciences, North-West University, Potchefstroom 2520, South Africa

**Keywords:** Polysulfone, ionomer, thiolation, sulfonation, proton exchange membrane, nucleophilic substitution

## Abstract

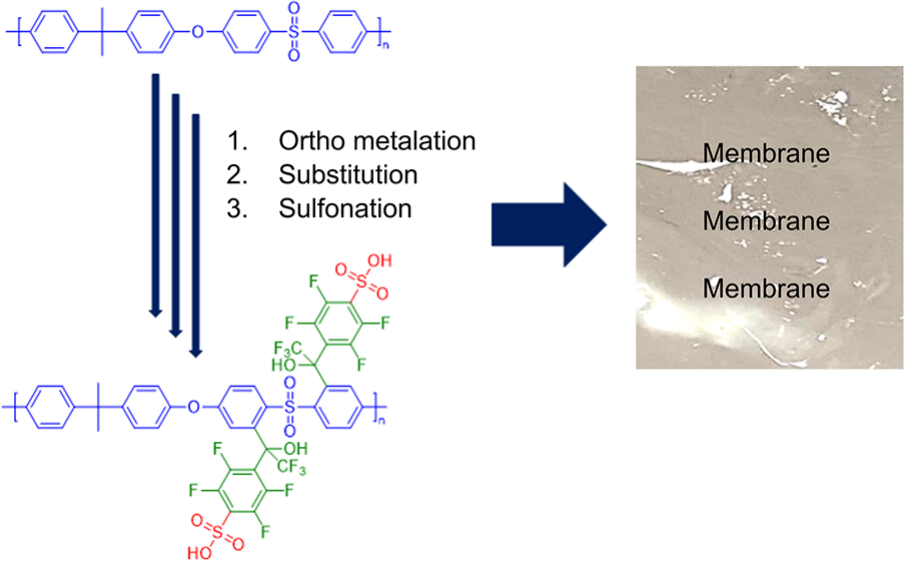

Polyethersulfone (PSU) as a commercially available polymer offers many different opportunities for functionalization for diverse fields of application, for example, electrophilic substitutions like sulfonation and bromination or nucleophilic reactions such as lithiation. This study presents three different polysulfone derivatives, first functionalized by a lithiation reaction, followed by a reaction with carbonyl compounds containing pentafluorophenyl groups. In the last step, the pentafluorophenyl moieties of the modified PSU were sulfonated by thiolation and subsequent oxidation to sulfonic acid groups. Those novel PSU derivatives were characterized by NMR, DSC, TGA, GPC, and titration. Based on these ionomers, we show the fabrication of pure and acid–base blend membranes with promising proton conductivities. These novel sulfonic acid groups containing materials are potentially promising candidates for membranes or ionomers in electrochemical applications such as proton exchange membrane fuel cells (PEMFCs), proton exchange membrane water electrolysis (PEMWEs), or redox flow batteries (RFBs).

Polyethersulfone (PSU) stands out as a highly utilized commercial polymer in the realm of medical applications, particularly prized for its role as a material for micro- and ultrafiltration membranes produced by phase inversion.^1^ One of its key advantages lies in its remarkable modifiability, achieved through various electrophilic reactions such as bromination,^2^ sulfonation,^3^ nitration,^4^ Friedel–Crafts acylation,^5^ as well as nucleophilic substitution reactions like lithiation followed by reaction with electrophiles.^6−8^

Cation-exchange PSU ionomers, encompassing phosphonated,^9^ sulfonated,^10^ and carboxylated^11^ variants, have garnered substantial attention as substitute materials for perfluorosulfonic acids (PFSAs) like Nafion, 3M, and Fumion in energy applications. These applications span across PEM (proton exchange membrane) fuel cells,^12^ PEM water electrolysis,^13^ and redox-flow batteries.^14^

However, the journey to enhanced performance encounters challenges, especially with nonfluorinated sulfonated PSU or other nonfluorinated sulfonated aromatic main-chain polymers such as poly(aryl ether ketone)s like sulfonated poly(ether ether ketone) (sPEEK)^15^ or sulfonated poly(dimethyl aryl ether) (sPPO).^16^ Some of these polymers exhibit inadequate proton conductivity^17^ and stability, particularly under elevated temperatures and/or reduced humidity conditions.^18^ This deficiency is attributed to the hydrolysis and thermal splitting-off sensitivity and insufficient acidity of the SO_3_H (sulfonic acid) group positioned directly on the aromatic backbone of PSU, mainly if the SO_3_H group is located in the aryl ether part of the PSU repeat unit.^19^ One concept to improve the properties of aromatic hydrocarbon cation-exchange polymers includes the synthesis of block-*co*-polymers consisting of hydrophobic and sulfonated hydrophilic blocks with the sulfonic acid group pendent to the main chain^20^ or a side chain^21^ of the hydrophilic block. Due to the nanophase separation between hydrophilic and hydrophobic blocks, these block copolymers show high proton conductivities already at low ion-exchange capacities and reduced humidity. As the ether linkages in aromatic polyethers are prone to radical attack, Kreuer et al. have synthesized an aromatic sulfonated polysulfone by polycondensation of sodium 5,5′-sulfonylbis(2-fluorobenzenesulfonate) with 4,4′-thiodibenzenethiol, followed by oxidation of the thio bridges, ending up in a polymer with high proton conductivity and excellent chemical stability^22^ Other polymers avoiding ether bridges are polymers prepared by hydroxyalkylation, One example is a recent study on the polyhydroxyalkylation of biphenyl with isatin, followed by sulfonation, where highly stable sulfonated ionomers were obtained which showed excellent proton conductivities of up to 200 mS/cm at 80 °C.^23^ Other approaches are different types of ionic liquids that are combined with other compounds, such as organic, inorganic, or inorganic–organic hybrid skeletons, possessing a combination of high ionic conductivity and a wide electrochemical stability window with low leaching percentage of the ionic liquid, see the review.^24^ In a recent study by Gubler et al., radiation-grafted sulfonated poly(a-methylstyrene) copolymerized with styrene moieties having a pendant crown ether-Cer(III) complex with Ce(III) as radical scavenger has been prepared and showed significant reduction of polymer degradation when applied in a fuel cell.^25^

In this work, the perfluoroaromatic side chain-modified PSU polymers were sulfonated via a nucleophilic route in a two-stage procedure. This two-stage process involved the thiolation with NaSH (sodium hydrosulfide), followed by the oxidation of the thiolated PSU polymers with H_2_O_2_ (hydrogen peroxide), a method intricately detailed in a prior study by Atanasov and Kerres.^26^ This approach opens new avenues for tailoring PSU polymers with perfluoroaromatic side chains to achieve optimal performance in diverse applications.

The starting materials were synthesized by a two-step, one-pot reaction. In the first step, the PSU was lithiated by *n*-BuLi at the ortho position to the SO_2_ bridge.^6^ Afterward, perfluoroacetophenone (a) or perfluorobenzophenone (b) or pentafluorobenzenesulfonylfluoride (s) were added as a nucleophile to the reaction mixture and reacted with the lithium-masked carbanion. Using this method, PSU polymers with three different side chains were obtained, abbreviated as PSUa, PSUb, and PSUs in the following.

For each polymer, the degree of substitution (DOS) was determined by ^1^H NMR spectroscopy (spectra are shown in SI). PSUa contains 1.60 side chains per repeating unit, PSUb 1.76, and PSUs 1.16 side chains. Theoretically, a maximum DOS of 2 is possible because the two aromatic rings pendant to the sulfone bridge of the PSU repeating unit became deactivated after the first deprotonation with *n*-BuLi and carbanion formation. In reality, the DOS of 100% (2 functional groups per repeating unit) was not reached. This is caused by one or more of the following issues during the nucleophilic substitution step.

On the one hand, the attached perfluorinated side group significantly reduces the solubility of the polymer in the solvent, which leads to polymer precipitation during the substitution reaction (mainly observed for PSUs). On the other hand, there is some sterical hindrance after the attachment of the first side group, leading to a lower probability of the attachment of a second side group.

To obtain ion-conducting polymers, ion-exchange functional groups need to be introduced into the three synthesized polymers. Hence, sulfonic acid groups were introduced via a two-step synthetic pathway as mentioned above.^26^ Within the first step, the fluorine atom in the para position of the side chain was substituted with a thiol group via a nucleophilic substitution reaction. In the second step, thiol was oxidized, resulting in a sulfonic acid group. Scheme 1 shows an exemplary reaction scheme for s-PSUa. The success of the sulfonation reaction for each of the three discussed PSU derivatives was proven by ^1^H- and ^19^F-NMR spectroscopy (Figure 1).

**Scheme 1:**
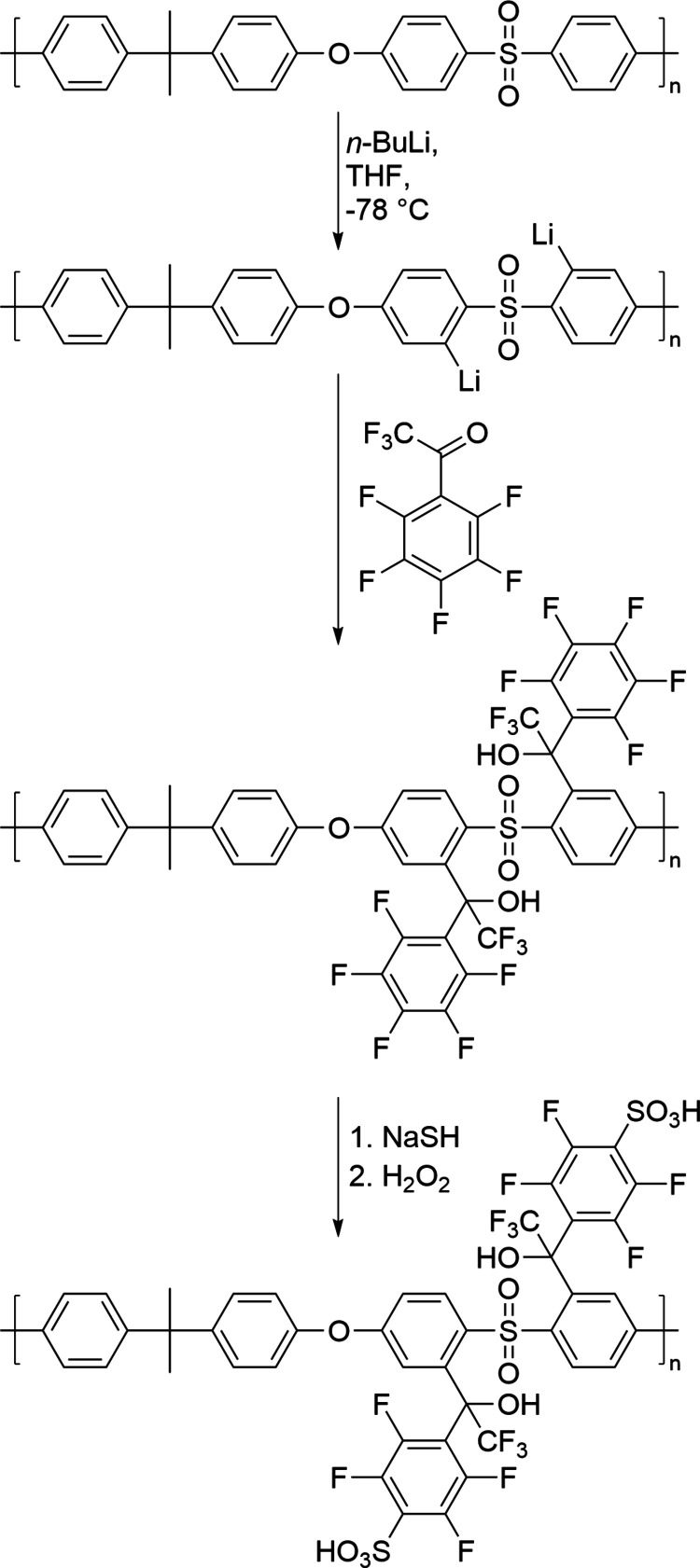
Scheme of the PSU Reaction Sequence Lithiation–Reaction with a Carbonyl Compound–Thiolation–Oxidation with the Example of Perfluoroacetophenone

**Figure 1:**
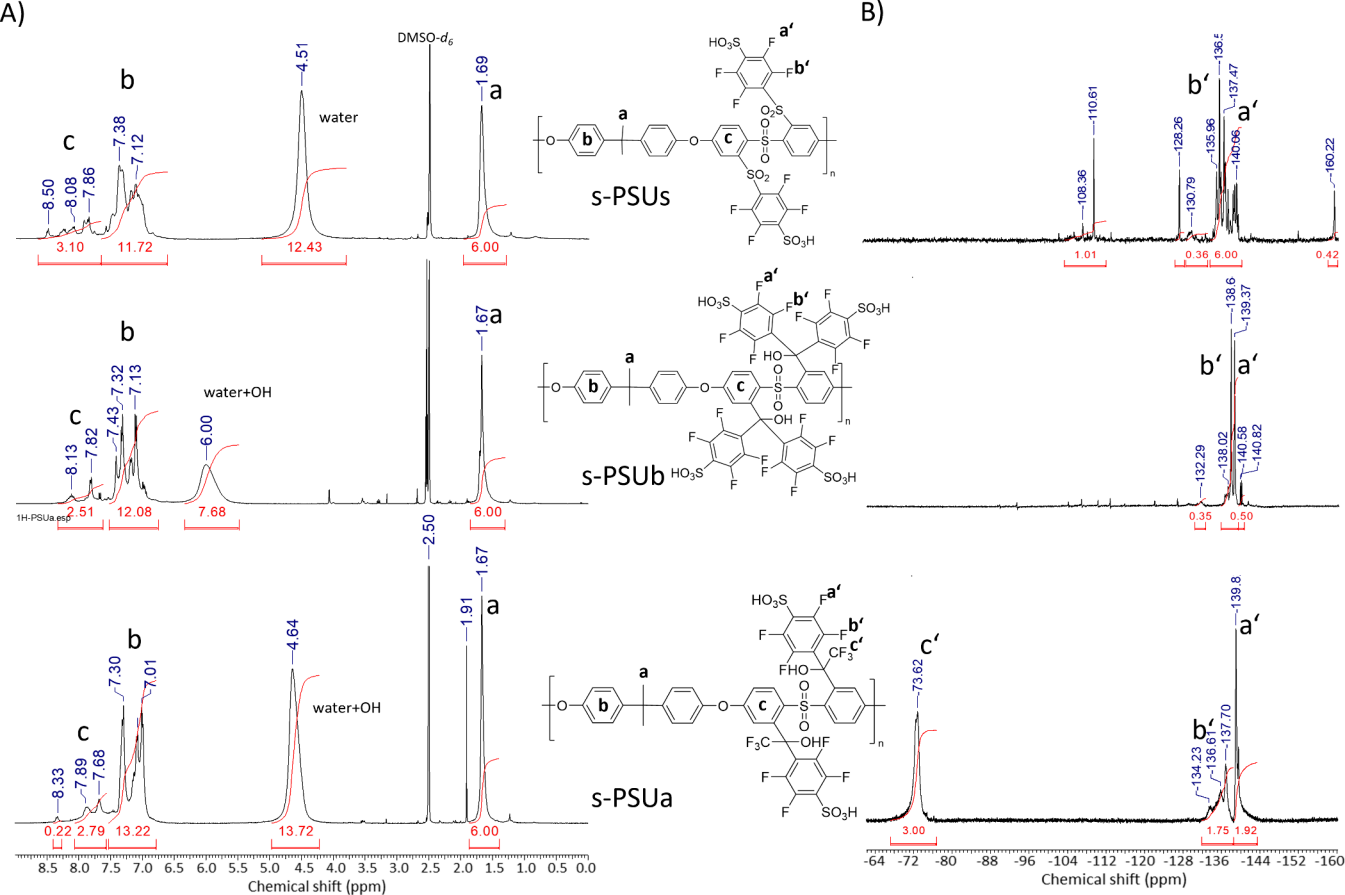
^1^H NMR (A) and ^19^F NMR (B) spectra of s-PSUa, s-PSUb, and s-PSUs in d-DMSO at room temperature.

In general, the sulfonation reaction should not significantly influence the protons’ chemical shift of the PSU main chain in the ^1^H NMR spectrum because no chemical reaction affects the main chain during the sulfonation step. The isopropylidene groups could be detected in the ^1^H NMR spectra of the side-chain perfluoro-modified and sulfonated PSUs at a chemical shift of 1.7 ppm (assigned as *a* in Figure 1A). The hydrogen atoms from the phenylene moieties are generally separated into two groups: Due to the electron-withdrawing effect of the attached fluorinated side chains, the signals of the main chain’s protons in direct neighborhood to them were shifted to the low field with a chemical shift in the range 7.7 and 8.5 ppm. These signals are marked with *c* in Figure 1A. The protons bound to the aromatic units in the main chain, apart from the fluorinated side groups, showed lower chemical shifts in the range between 7.0 and 7.5 ppm. These signals are assigned as *b* in Figure 1A.

The ^19^F-NMR-spectrum (Figure 1B) indicates successful sulfonation of the para position of the fluorinated phenyl rings in the side chains. Only the signals for the fluorine groups in meta (a′) and ortho (b′) positions were detected in the corresponding spectra. If the side chain contained a CF3-group (s-PSUa), an additional signal c′ at −73.6 ppm was detected.

In addition to the polymers’ structural properties, their thermal properties, are essential for their potential field of application. Therefore, the thermal properties of the modified PSUs were determined by DSC and TGA–FTIR-coupling.

The TGA-FTIR coupling method enables the determination of thermal properties and the characterization of degradation products in a single experiment. While the thermal properties of the samples will be investigated using a TGA measurement, the examined sample will be degraded to gaseous and solid residues. The gaseous decomposition products were further characterized by FTIR spectroscopy.^27^

Commercially available PSU is thermally stable up to 500 °C, which is well-known from literature.^28−30^Figure 2 shows the TGA traces of the substituted and sulfonated PSU derivatives and the corresponding Gram Schmidt traces. The FT-IR spectra of the TGAs gas outlet stream are shown in SI in Figures S8–S10. Each sulfonated polymer showed three steps in the TGA profile: The first step between room temperature (RT) and 150 °C is due to the evaporation of residual water and solvent. No water or solvent evaporation was detectable in the Gram Schmidt trace plot, representing the entire FT-IR signal intensities of the thermally split-off low-molecular moieties. The second step, between 250 and 400 °C, is caused by the polymers’ desulfonation. For the second degradation step (desulfonation), the Gram Schmidt signal became essential. The FT-IR spectra of the exhaust gases showed a valence vibration for SO_2_ at about 1300 cm^–1^ starting from 250 up to 400 °C. This signal can be assigned to the desulfonation of the polymers. In the last step, the polymer backbone itself degrades. This happened in the temperature range between 400 and 550 °C. In this temperature area, the Gram Schmidt signal increased significantly due to the entire decomposition of the polymer backbones.

**Figure 2:**
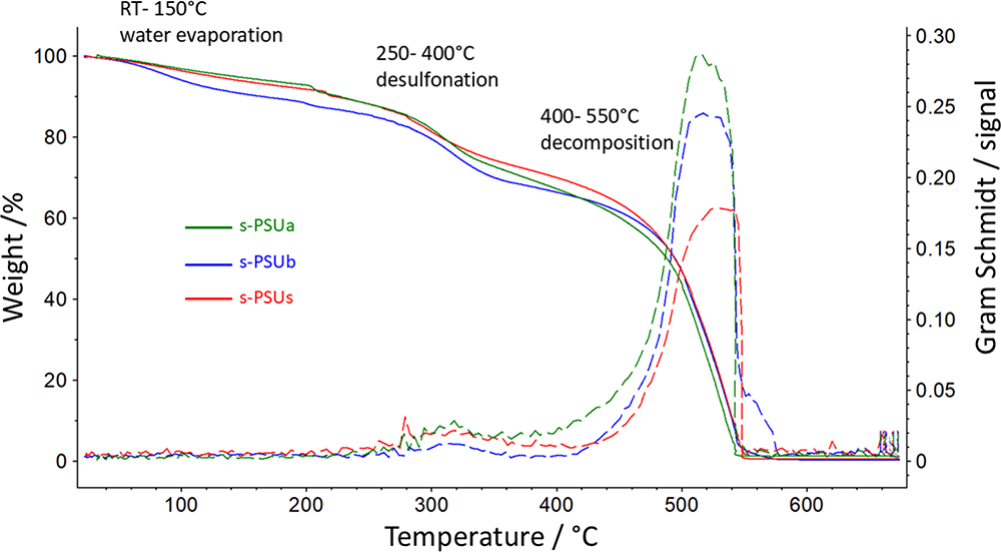
TGA curves and the corresponding Gram-Schmidt trace plots of s-PSUa, s-PSUb, and s-PSUs.

The polymers’ decomposition was detected by the appearance of a valence vibration of CO and CO_2_ at 2200 and 2300 cm^–1^, respectively, at temperatures above 400 °C. It is worth noting the appearance of HF (hydrofluoric acid) at temperatures above 400 °C. This is detectable due to the defluorination of the polymers’ fluorinated side chains.

To investigate the influence of the sulfonated side chains on the glass transition temperature T_g,_ the second DSC heating curve of unmodified PSU was compared to those of the sulfonated PSU derivatives. The second heating and cooling cycle was used due to the evaporation of residual water and solvents and polymer phase “history” during the first heating and cooling cycle. The DSC trace of virgin PSU shows a *T*_g_ of 189 °C, comparable to the literature (Figure S12).^29^ The sulfonated PSU derivatives show a lower glass transition temperature than virgin PSU. For s-PSUa, a *T*_g_ of 160 °C; for s-PSUb, a *T*_g_ of 154 °C; and for s-PSUs, a *T*_g_ of 150 °C was determined. Compared to unmodified PSU, a higher glass transition temperature was noted for PSU derivatives, which are sulfonated in the main chain.^31,32^ In this study, we show a decrease in *T*_g_ by introducing fluorinated side chains and their sulfonation.

The molecular weight distributions of the three sulfonated polymers were determined by GPC measurements in NMP (1-methyl-2-pyrrolidone) as the eluent. All three substituted and sulfonated PSUs still show a high molecular weight. s-PSUa showed a mean molecular weight of 33 000 g mol^–1^ with a polydispersity (PDI) of 1.88. s-PSUb has a molecular weight of 22 500 g mol^–1^ and a PDI of 2.20. For s-PSUs, a molecular weight of 41 600 g mol^–1^ and a PDI of 3.75 was determined (Figure S13).

Finally, the sulfonated PSU derivatives’ ion exchange capacities (IEC) were determined by an acid–base titration with a sodium hydroxide solution. Due to the incomplete substitution with perfluoroaromatic side chains, the theoretical IEC differs from the one expected by the chemical structure. Hence, the theoretical values for IEC_direct_ were calculated using eq 1, considering the determined DOS.
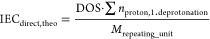
1For s-PSUa, an IEC_direct_ of 1.15 mmol g^–1^ was titrated compared to an IEC_direct,theo_ of 1.46 mmol g^–1^. For s-PSUb, an IEC_direct_ of 1.57 mmol g^–1^ in comparison to an IEC_diret,theo_ of 2.44 mmol g^–1^, and for s-PSUs an IEC_direct_ of 1.09 mmol g^–1^ in contrast to an IEC_diret,theo_ of 1.10 mmol/g. The titrated IEC of s-PSUs is in the same range as the theoretical one. However, the experimental IECs for s-PSUa and s-PSUb differ significantly from those calculated. On the one hand, this could be caused by an incomplete thiolation of para F in the side functional group, although a complete substitution was indicated by NMR-spectroscopy. On the other hand, an incomplete oxidation of the thiol to a sulfonic acid group also cannot be excluded. Furthermore, nonaccessible domains during the ion exchange could explain the difference in the calculated IEC_theo_. The polymer structure of the polymers shown here is more rigid than Nafion’s highly flexible polymer chain.

The modified PSUs were cast into membranes to make the promising ionomers available for potential applications like PEMFC, PEMWE, or RFB. For higher IECs > 2.0 mmol g^–1^, arylene main-chain ionomers, such as sPSU sulfonated in the main chain, are often water-soluble.^19^ In the case of water-solubility of the modified ionomers, they were blended with a polybase (e.g., a polybenzimidazole such as OPBI) to obtain water-insoluble acid–base blend membranes.^33−35^ From the (water-insoluble) two PSU derivatives with a lower IEC (relating to the number of available reaction sites for sulfonation), s-PSUa and s-PSUs, it was possible to directly cast membranes from DMSO solutions. The water-soluble s-PSUb was blended with OPBI (80 wt % s-PSUb and 20 wt.% OPBI). All three casted membranes had a thickness of around 20 μm. Photographs of the three different membranes are shown in Figure 3.

**Figure 3:**
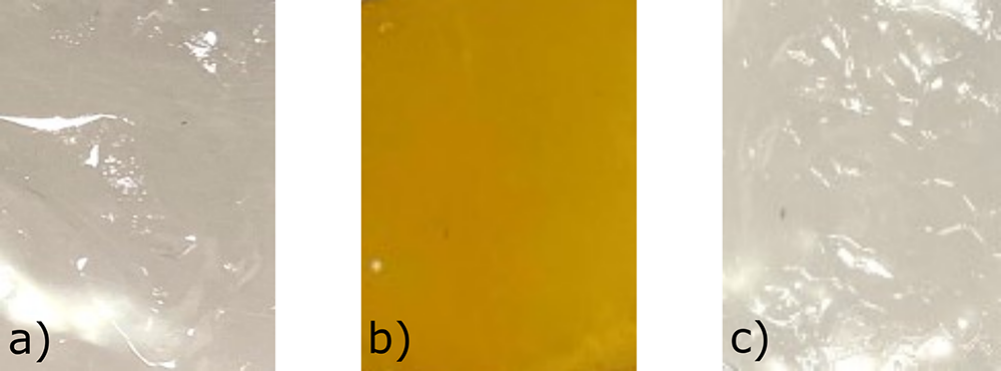
Photographs of two pure membranes and one blend membrane: (a) s-PSUa, (b) s-PSUb (80 wt %)/OPBI (20 wt %), and (c) s-PSUs.

Furthermore, the essential characteristics of the three membranes were evaluated. Their ionic conductivity was measured by impedance spectroscopy. Moreover, the water uptake and swelling in water were determined. Furthermore, their stability in Fenton’s reagent was evaluated. The corresponding data are summarized in the following Table 1. The determined values for dimensional swelling of the membranes are shown in SI (S-Table 1).

**Table 1:** Listed Values for the Prepared Membranes: Thickness d, Ionic Conductivity σ (Measured in 0.5 M H_2_SO4), Water Uptake WU, Swelling in Thickness Δd, and Stability in Fenton’s Reagent

Sample	*d*, μm	IEC_direct,theo_, mmol·g^–1^	IEC_direct_, mmol·g^–1^	σ, mS·cm^–1^	WU_25°C_, %	WU_85°C_, %	Δ*d*_25°C_, %	Δ*d*_85°C_, %	Fenton’s_25°C_, %
s-PSUa	24 ± 2.0	1.46^a^	1.15^a^	100 ± 14^b^	26.0	40.2	30.1	36.5	86.3
s-PSUs	18 ± 0.5	1.10^a^	1.09^a^	52 ± 12^b^	18.9	38.7	3.9	10.0	95.3
s-PSUb/OPBI (IEC = 0.8)	17 ± 1.9	2.44^a^	1.57^a^	55 ± 19^b^	16.3	16.4	10.1	11.0	91.4

a IEC of the pure polymer.

b Measured in 0.5 M H_2_SO_4_.

All water-insoluble membranes displayed sufficient conductivities. The pure membrane of s-PSUa possessed a conductivity of 100 mS cm^–1^, and the pure membrane of s-PSUs showed a conductivity of 52 mS cm^–1^. Due to the water solubility of s-PSUb, only blend membranes with OPBI were stable enough to determine their ionic conductivity. The blend membrane, which had a theoretical IEC of 0.8 mmol g^–1,^ showed a conductivity of 55 mS cm^–1^. Compared to each other, s-PSUa displayed the highest ionic conductivity, which was expected due to the high IEC. However, blending polyacids with polybases lowers the IEC; the blend membrane of 80 wt % s-PSUb and 20 wt % OPBI showed sufficient ionic conductivity for their potential field of application, such as fuel cells or electrolysis. Remarkably, all membranes exhibited a low to acceptable water uptake (WU) at room temperature and 85 °C. In addition, they exhibited sufficient ionic conductivity. Their dimensional swelling Δd is acceptable, as well. However, s-PSUa shows a higher dimensional swelling compared to that of the other two membranes. Furthermore, all three membranes showed good stability in Fenton’s reagent at room temperature over 8 h.

Within this study, we present a new group of sulfonated PSU derivatives. We were able to sulfonate all introduced side chains (perfluoroacetophenone, perfluorobenzophenone, and pentafluorobenzensulfonylfluoride), which all contain pentafluorophenyl groups. They could be successfully characterized by NMR spectroscopy. All three materials show appropriate thermal stabilities up to 250 °C. In addition, they have high ion exchange capacities between 1.09 and 1.57 mmol g^–1^, which leads to good solubility in polar solvents.

Membranes and blend membranes from these novel sulfonated polymers show promising high conductivities and mechanical properties for their potential field of application. Furthermore, the membranes possess low water uptake and low swelling.

In ongoing work, it is planned to introduce additional side chains to enhance the material properties regarding proton conductivity, solubility, and thermal and mechanical stability.

## Supporting Information Available

Complete experimental details and methods used; NMR spectra of the nonsulfonated PSU derivatives; FTIR spectra of the TGA coupling measurements; TGA traces of unmodified and modified PSUs compared to sulfonated ones; DSC measurements of the three sulfonated PSUs; corresponding GPC curves; reaction schemes to obtain s-PSUb and s-PSUs; in-plane swelling data of the three membranes that were produced; ionic conductivities of different PSU-based or similar materials compared to the three membranes produced by this study (PDF)
