# Supplementary material for: Synthesis and Characterization of Novel Perfluoro Aromatic Side Chain Sulfonated PSU Ionomers
Source: ACS Polym Au. 2024 Oct 14;4(6):492–7. doi: 10.1021/acspolymersau.4c00059 (PMC11638786; doi:10.1021/acspolymersau.4c00059)
Supplement: Supplementary file 1 — lg4c00059_si_001.pdf [file lg4c00059_si_001.pdf]

## Supporting Information

# Synthesis and characterization of novel perfluoro aromatic side chain-sulfonated PSU ionomers

Philipp Martschin,*[a,b] Vladimir Atanasov,[c] Simon Thiele [a,b] and Jochen Kerres *[a,d]

*Corresponding authors

## Materials and Methods

### Materials

Polysulfone Udel® (supplier: Solvay S. A.), respectively, was used as received. Sodium hydrosulfide hydrate ($NaSHxH_2O$) (Sigma-Aldrich) was used as received. Tris(trimethylsilyl)phosphite, perfluoro-acetophenone, perfluorobenzophenone, and pentafluoro-benzenesulfonylfluoride were purchased at Manchester Organics. *n*-butyllithium (2.5 M in Hexanes), THF (dry, 250 ppm butylhydroxytoluene as inhibitor), heptane, and other solvents were purchased at Sigma Aldrich. OPBI was purchased at Fumatech. All chemicals were used without further purification steps. The glassware was cleaned and air-dried before use. All syntheses were carried out under an argon atmosphere.

### Instruments, polymer, and membrane characterization

The chemical composition and structure of starting materials and products were characterized by Proton, Fluorine, and Phosphor NMR-spectroscopy. The $^1H$ and $^{19}F$ spectra were recorded at room temperature by a 500 MHz JEOL ECX-400 NMR spectrometer and a Bruker Avance 400 MHz NMR spectrometer.

The molecular weight distribution for the non-sulfonated reaction products of lithiated PSU with different perfluoroaromatic compounds ($M_n$, PDI) was determined using gel permeation chromatography (GPC). Therefore, a GPC system SECurity² from PSS Polymer Standard Service Mainz, Germany, equipped with a UV and refractive index detector, was used. The chromatography was done with a PSS SDV LUX GUARD, a PSS SDV LUX 3 µm 1000 Å, and a PSS SDV LUX 3 µm 10000 Å column set, which were calibrated with a series of polystyrene standards in THF. The respective polymer was dissolved in non-stabilized THF with a concentration of 1 g/L for the measurement itself. The polymer solution was filtered by a syringe filter over a microporous PTFE membrane (0.45 µm), injected, and separated over the columns with a 1 mL/min flow rate.

The molecular weight distribution for the sulfonated compounds ($M_n$, PDI) was determined using gel permeation chromatography (GPC). Therefore, a GPC system SECurity² from PSS Polymer Standard Service Mainz, Germany, equipped with a UV and refractive index detector, was used. The chromatography was done with a PSS GRAM GUARD, a PSS GRAM 10 µm 100 Å, and a PSS GRAM 10 µm 3000 Å column set, which were calibrated with a series of polystyrene standards in NMP. The eluent was mixed with 1 g/L LiBr to shield the charged groups of the polymer. The respective polymer was dissolved in the prepared NMP with a concentration of 1 g/L for the measurement itself. The polymer solution was filtered by a syringe filter over a microporous PTFE membrane (0.45 µm), injected, and separated over the columns with a 1 mL/min flow rate.

The thermal stability of the synthesized polymers was determined by TGA measurements. For that purpose, a TGA 800 from Perkin Elmer and STA 499C (NETZSCH TGA) coupled with an FT-IR spectrometer (Bruker) were used. The decomposition measurements were performed with a 10 K/min heating rate under a synthetic air (SA) atmosphere, starting from room temperature to 900 °C.

To determine the glass transition temperature ($T_g$) of the synthesized polymers, a DSC 3+ from Mettler Toledo was used. The sample was heated and cooled down in two cycles, starting from room temperature to 300 °C with a heating/cooling rate of 20 K/min.

The ion exchange capacities (IECs) were determined by acid-base titration with an OMNIS Titrator from Metrohm. For the titration itself, the polymers/membranes in $H^+$ form were stirred in a warm, saturated sodium chloride solution to exchange the protons for sodium cations. The so exchanged $H^+$ ions were titrated with a 0.1 M sodium hydroxide solution to the equivalent point to determine $IEC_{direct}$.

$$IEC_{direct} = \frac{\Delta V_{NaOH} \cdot c_{NaOH}}{m_{sample}} \quad (1)$$

Then, a defined volume of 0.1 M sodium hydroxide solution was added. Afterwards, the solution was back titrated with 0.1 M HCl to determine $IEC_{total}$.

$$IEC_{total} = \frac{V_{NaOH,excess} \cdot c_{NaOH} - [(\Delta V_{NaOH} \cdot c_{NaOH}) + (\Delta V_{HCl} \cdot c_{HCl})]}{m_{sample}} \quad (2)$$

The polymer's/membrane's ionic conductivity was measured by impedance spectroscopy with a Zennium X workstation from Zahner, Kronach, Germany. The measurements were done at room temperature in 0.5 M sulphuric acid to minimize contact resistances according to a method described by Kerres *et al.* [1]. The ionic conductivity was calculated according to the following formula.

$$\sigma = \frac{d}{R \cdot A} [\mathrm{mS \cdot cm^{-1}}] \quad (3)$$

The samples' water uptake was determined at RT and 85 °C. The samples were dried in an oven and weighed in a dry state ($m_{dry}$). Afterward, the samples were placed in a vial with water and stored for 48 hours at RT and 85 °C. Then, the samples were taken out of the water, quickly dry wiped, and weighed in the wet state ($m_{wet}$). The water uptake was calculated based on the two determined values according to the following equation.

$$WU{:}\, \Delta m[\%] = \frac{m_{wet} - m_{dry}}{m_{dry}} \cdot 100[\%] \quad (4)$$

The samples' swelling in water was determined at RT and 85 °C. The samples were dried in an oven, and their thickness was measured in a dry state ($d_{dry}$). Subsequently, the samples were placed in a vial with water and stored for 48 hours at RT and 85 °C. Then the samples were taken out of the water, quickly dry wiped, and the thickness was measured in the wet state ($d_{wet}$). According to the following equation, the swelling ratio was calculated based on the two determined values.

$$Swelling\ Ratio{:}\, \Delta d[\%] = \frac{d_{wet} - d_{dry}}{d_{dry}} \cdot 100[\%] \quad (5)$$

The chemical stability, especially against radicals, was investigated by storing the samples in Fenton's reagent (4 ppm $Fe^{2+}$, 3 % $H_2O_2$) at 85 °C for 48 h. The samples were weighed before and after the test to determine a potential loss in mass.

To determine the membranes' swelling in x- and y-direction, the membranes were cut into defined pieces, and their length was measured before the test (BOT) was started ($l_{x-direction,BOT}$ and $l_{y-direction,BOT}$). Then, the membrane pieces were stored in water at room temperature and 85 ° C for three days. After three days (EOT), their length in x- and y- directions was measured again ($l_{x-direction,EOT}$ and $l_{y-direction,EOT}$). According to the following equations 6 and 7, the increase in size was calculated.

$$\Delta l_{x-direction} = l_{x-direction,EOT} - l_{x-direction,BOT} \quad (6)$$

$$\Delta l_{y-direction} = l_{y-direction,EOT} - l_{y-direction,BOT} \quad (7)$$

## Polymer modification syntheses

The starting material, Polysulfone (PSU), was entirely dissolved in dry THF under an Argon atmosphere at room temperature and subsequently cooled down to -78 °C. *n*-butyllithium (*n*-BuLi) was added dropwise to the solution until all water traces were removed, as indicated by reddish coloring. In a second step, two equivalents of *n*-BuLi per repeating unit PSU were added dropwise to the solution, and its color turned red[2]. After an hour, the respective electrophilic compound (perfluoroacetophenone, perfluorobenzophenone, or pentafluorobenzenesulfonylfluoride) was added in molar excess (6 mol of carbonyl per PSU repeating unit). The solution was left stirring at -78 °C until homogenized, and then the cooling bath was slowly warmed to room temperature overnight by switching off the thermostat. Subsequently, the polymer was precipitated in methanol and washed with methanol. Finally, the obtained colorless solid was dried under a vacuum.

## Sulfonation of PSUa

*Thiolation:* Polymer PSUa (5 g, 5.2 mmol pentaflurophenyl, 1 equiv.) was dissolved in DMAc (100 g), and sodium hydrosulfide hydrate ($NaSHxH_2O$) (1.45 g, 15.5 mmol, 3 equiv.) was added at RT under argon. The mixture was stirred at RT overnight (18 hrs). The next day, the reaction mixture was precipitated in water (0.5 L). Then conc. $H_2SO_4$ (3 ml) was stirred at RT for 10 minutes. The solid was filtered off and rinsed with deionized water.

*Oxidation:* In an ice bath, peracetic acid (30 %, 10 ml) and 3 drops of H2SO4 (98%) were cooled down to 0°C. Then, the solid (from the former step) was slowly added, stirred for 30 min at 0°C, and slowly heated to RT. The mixture was stirred at RT overnight (18 hrs). The next day, the reaction mixture was heated to 40 °C for 2 hrs, 60 °C for 2 hrs, and 80 °C for 1 hr. The mixture was then dialyzed (MWCO 12 kDa) in MiliQ water for 3 days and dried in a convection oven at 90 °C for 24 hrs and in a vacuum at 120 °C for 2 hours. Yield: 5.3 g (94%). The yields for the rest of the polymers were: s-PSUb 74%; and s-PSUs 84%.

## Membrane preparation

The polymers were cast as pure or blended membranes for application and measuring conductivity. The sulfonated polysulfones (s-PSUa, s-PSUb, and s-PSUs) were dissolved in DMSO for membrane preparation. Afterward, the viscous ionomer solution was cast onto a glass plate, doctor bladed, heated to 80 °C, and the solvent was evaporated in a convection oven. To ensure complete protonation, the membranes were post-treated by immersing them in a 10 wt.-% sulphuric acid at 85 °C for 96 h and washing them several times with water at 85 °C until a constant water pH-value was reached.

The sulfonated polysulfone (s-PSUb) and the blend component (OPBI) were dissolved separately in the same solvent for blend membrane preparation. Afterward, the polyacid was neutralized with triethanolamine to avoid crosslinking. Subsequently, the neutralized poly acid solution was mixed with the poly base until a homogeneous mixture was obtained. Afterward, the blend solution was cast onto a glass plate, doctor-bladed, heated to 80 °C, and then the solvent was evaporated in a convection oven. To ensure complete protonation, the membranes were post-treated by immersing them in 10 wt.-% sulphuric acid at 85 °C for 96 h and washing several times with water at 85 °C until a constant water pH-value was reached.

## Additional data

### NMR-spectra

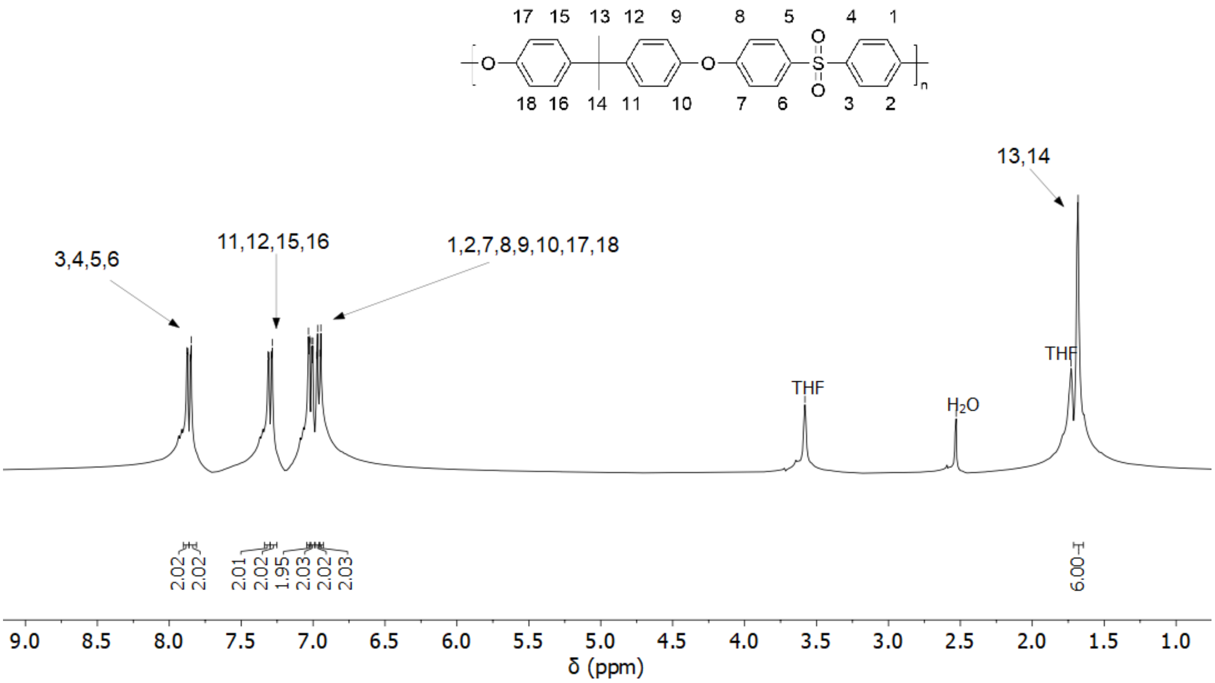


*Figure S1 – $^1$H-NMR spectrum of unmodified PSU, recorded at room temperature in d-THF.*

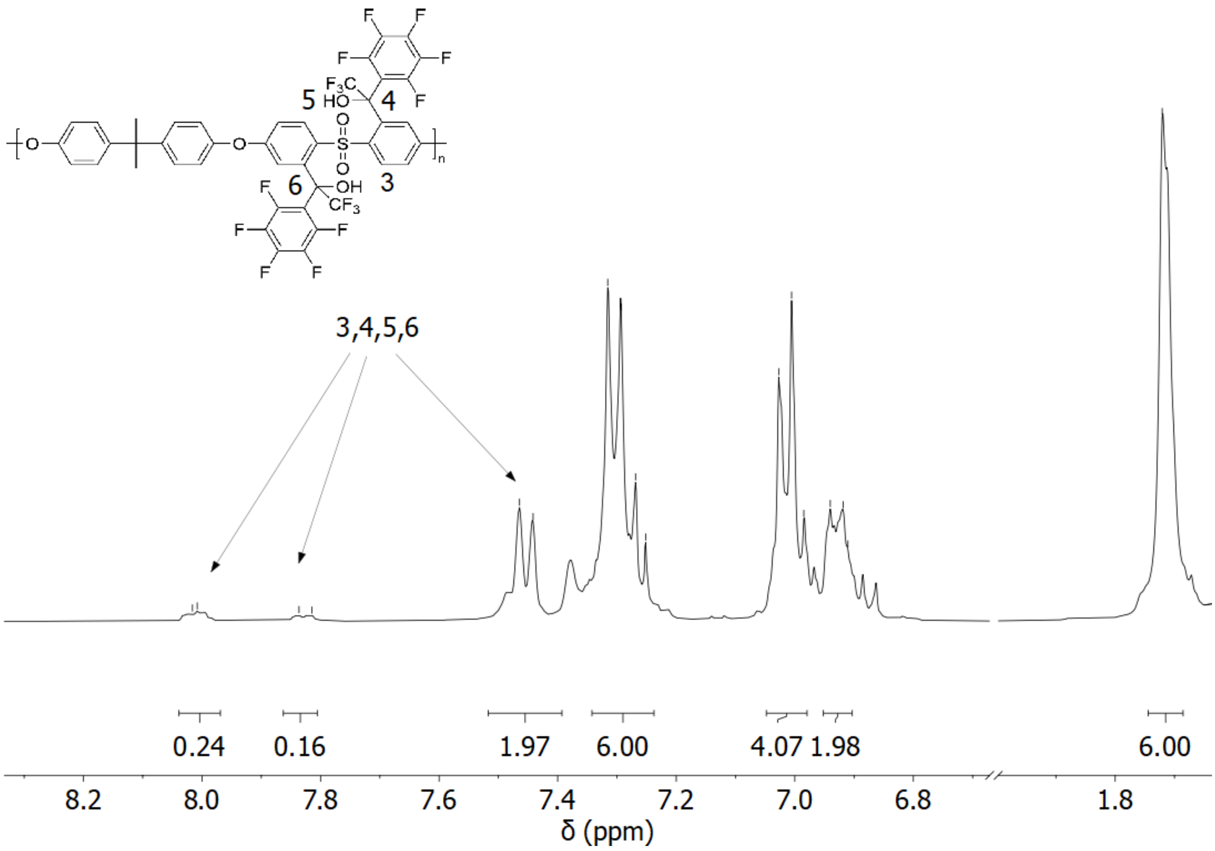


*Figure S2 – $^1$H-NMR spectrum of PSUa, recorded at room temperature in d-THF.*

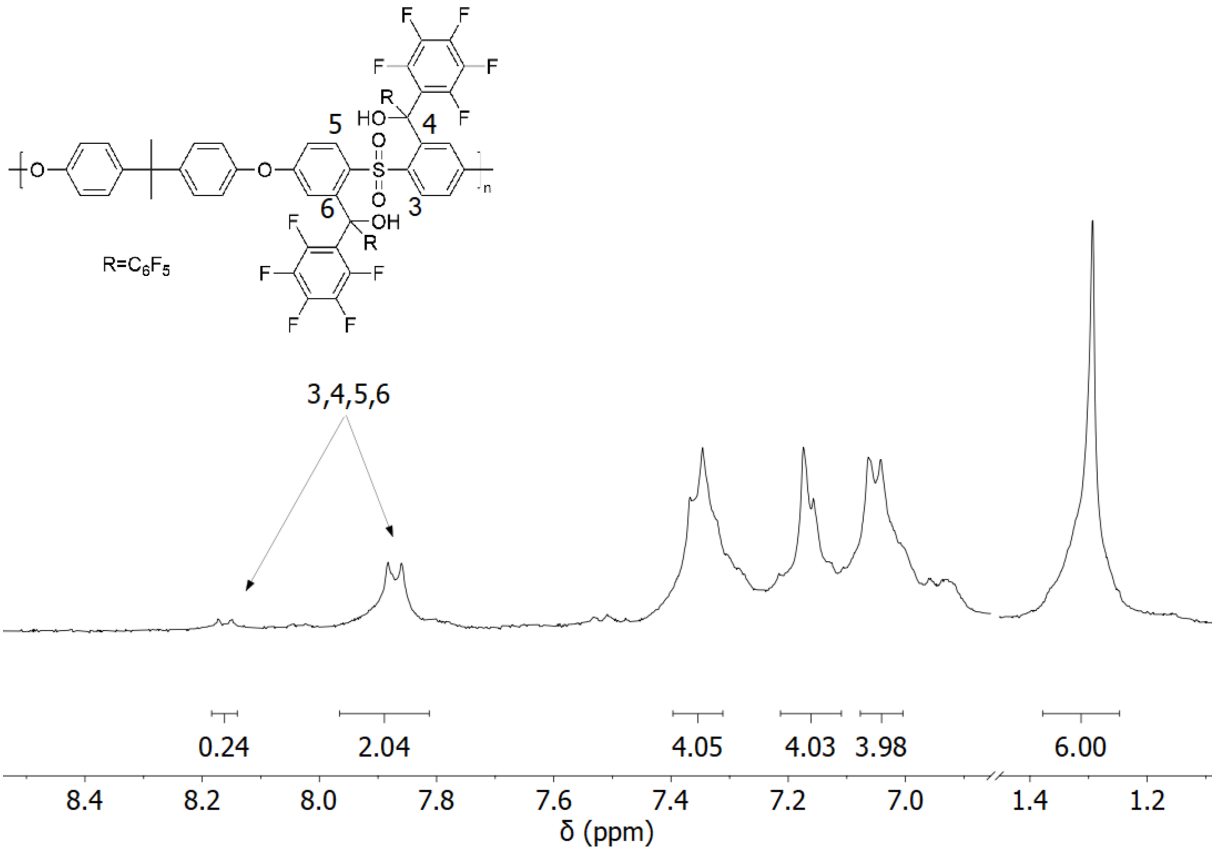


*Figure S3 – $^{1}$H-NMR spectrum of PSUb, recorded at room temperature in d-THF.*

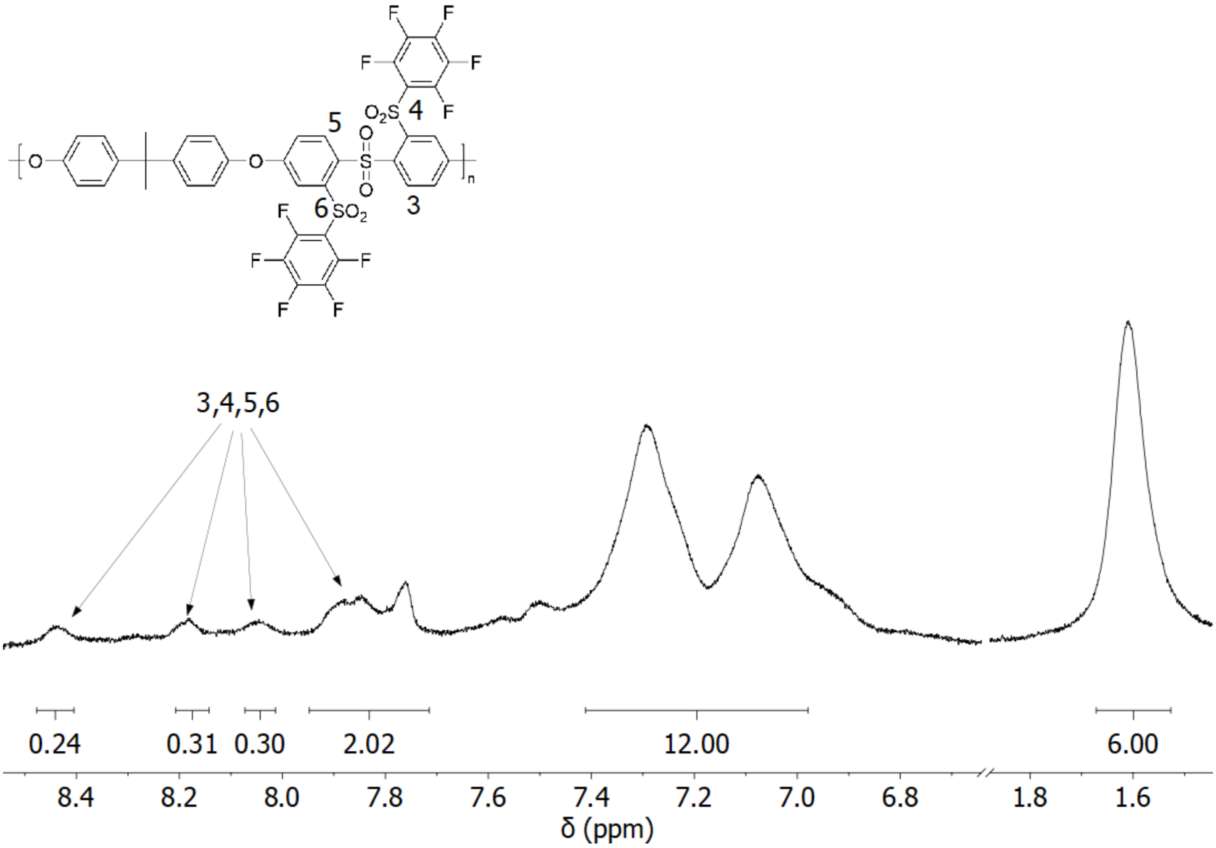


*Figure S4 – $^{1}$H-NMR spectrum of PSUs, recorded at room temperature in d-THF.*

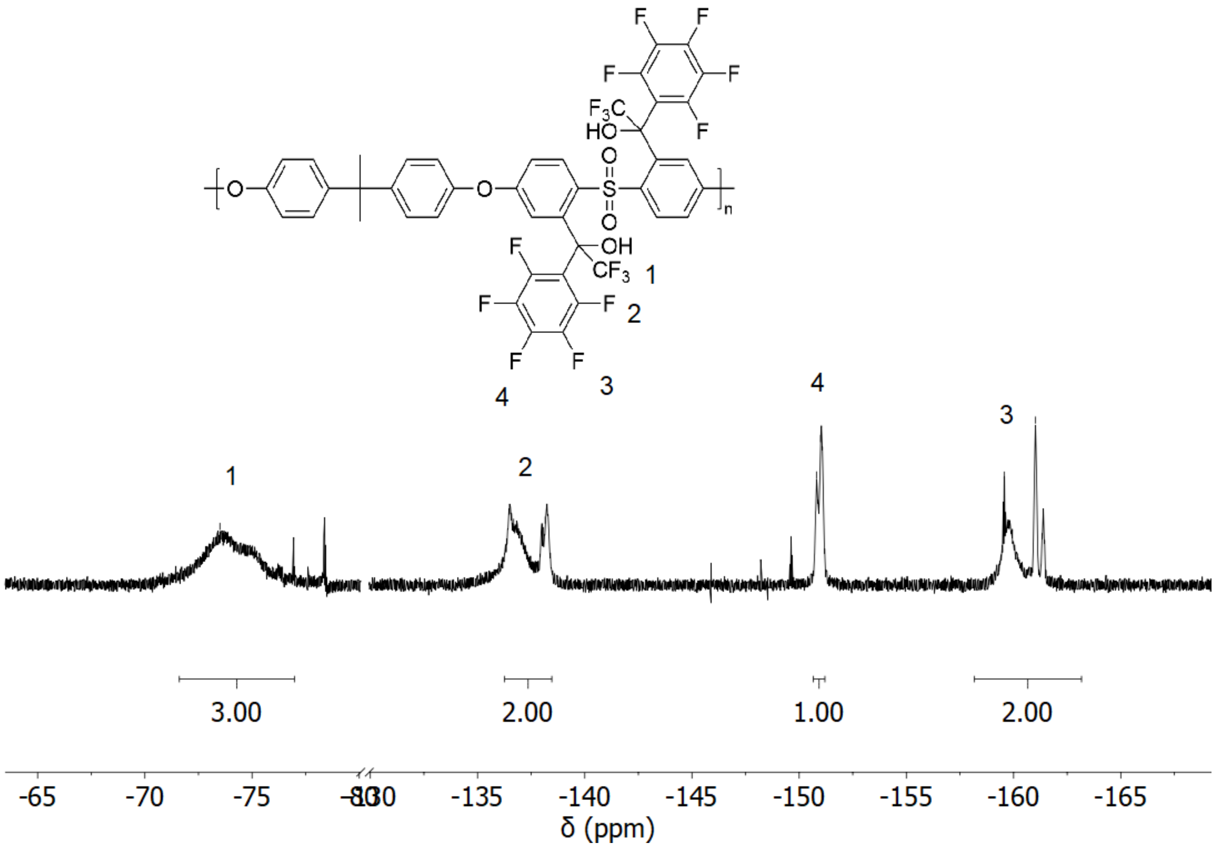


*Figure S5 – $^{19}$F-NMR spectrum of PSUa, recorded at room temperature in d-THF.*

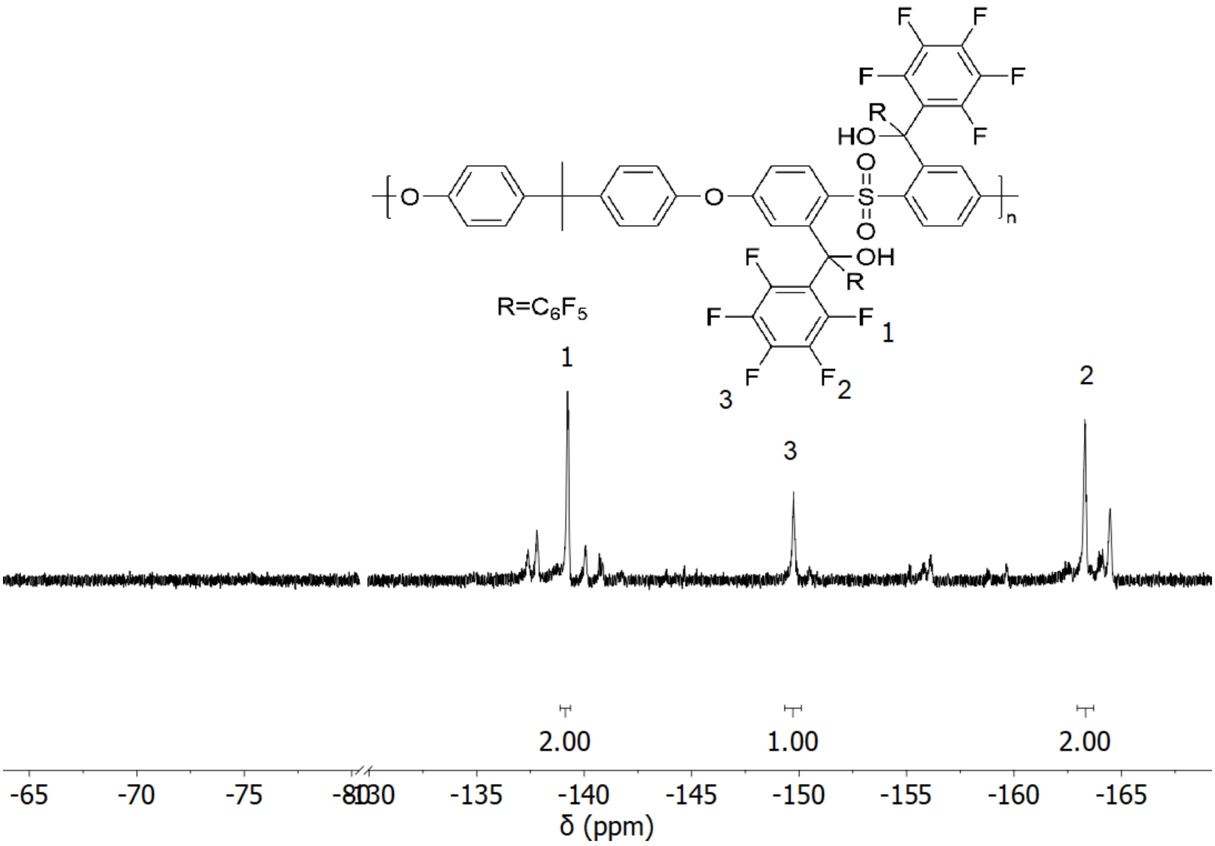


*Figure S6 – $^{19}$F-NMR spectrum of PSUb, recorded at room temperature in d-THF.*

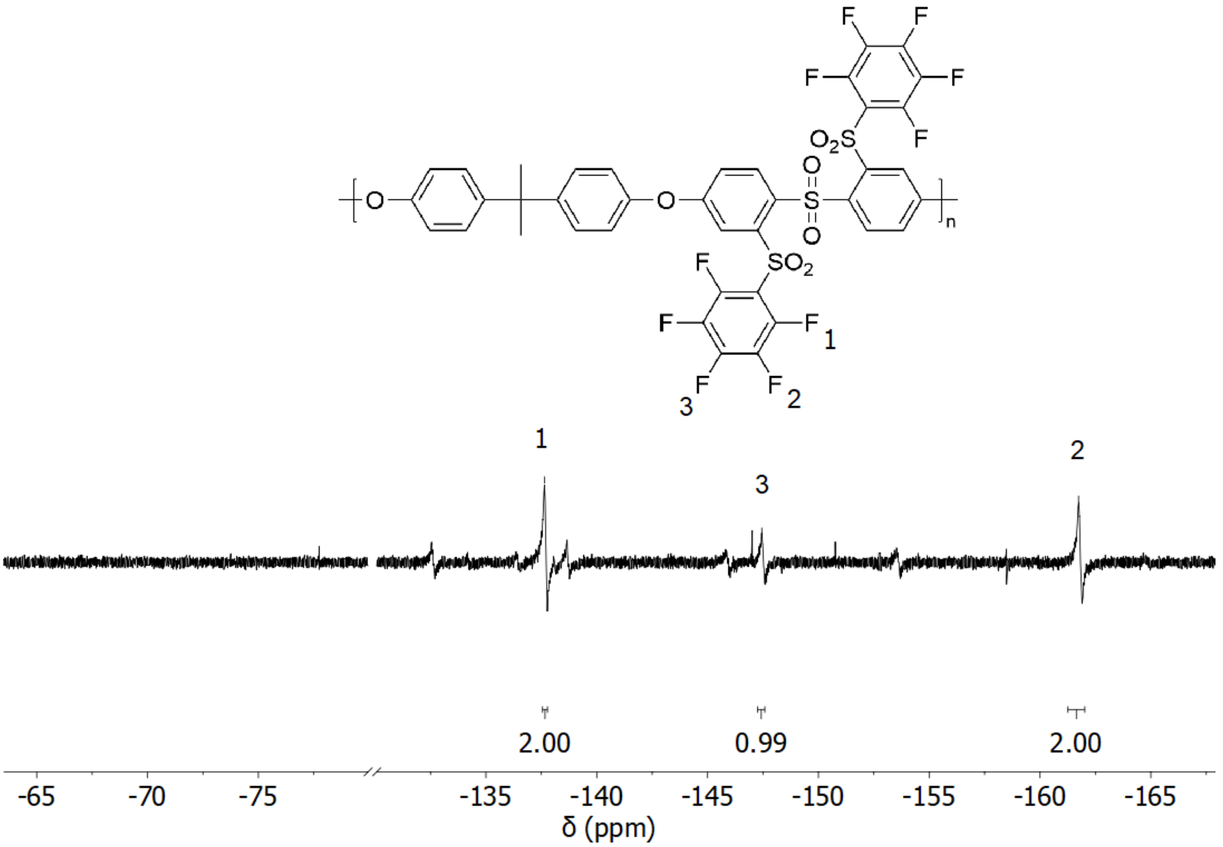


*Figure S7 – $^{19}$F-NMR spectrum of PSUs, recorded at room temperature in d-THF.*

## FTIR-spectra TGA coupling

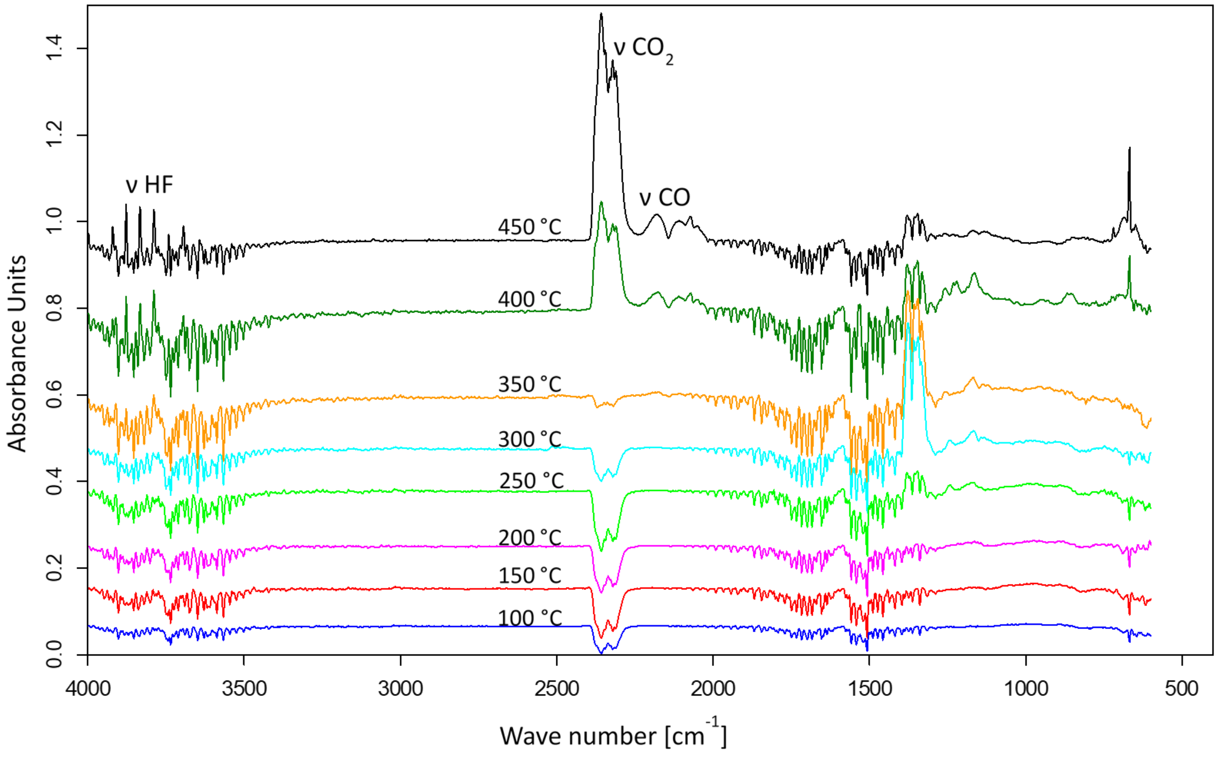


*Figure S8 – FTIR spectra of the gas outlet streams from the TGA measurement of s-PSUa.*

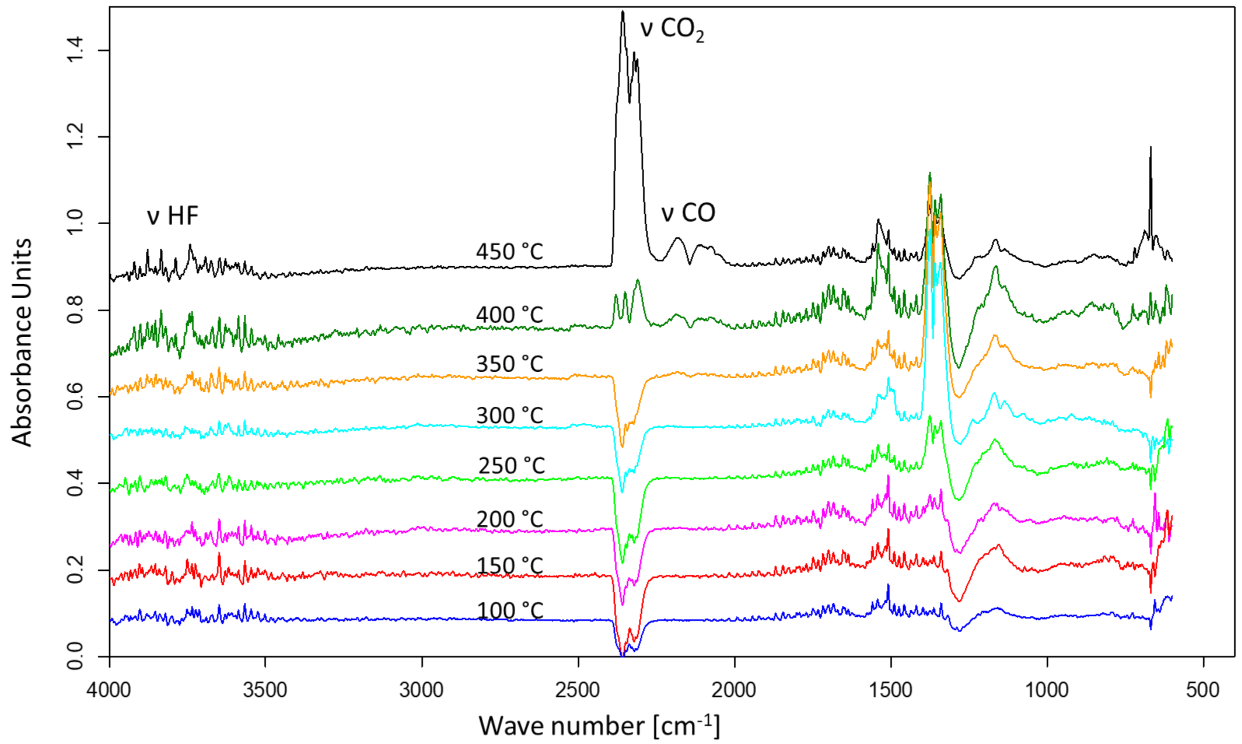


*Figure S9 – FTIR spectra of the gas outlet streams from the TGA measurement of s-PSUb.*

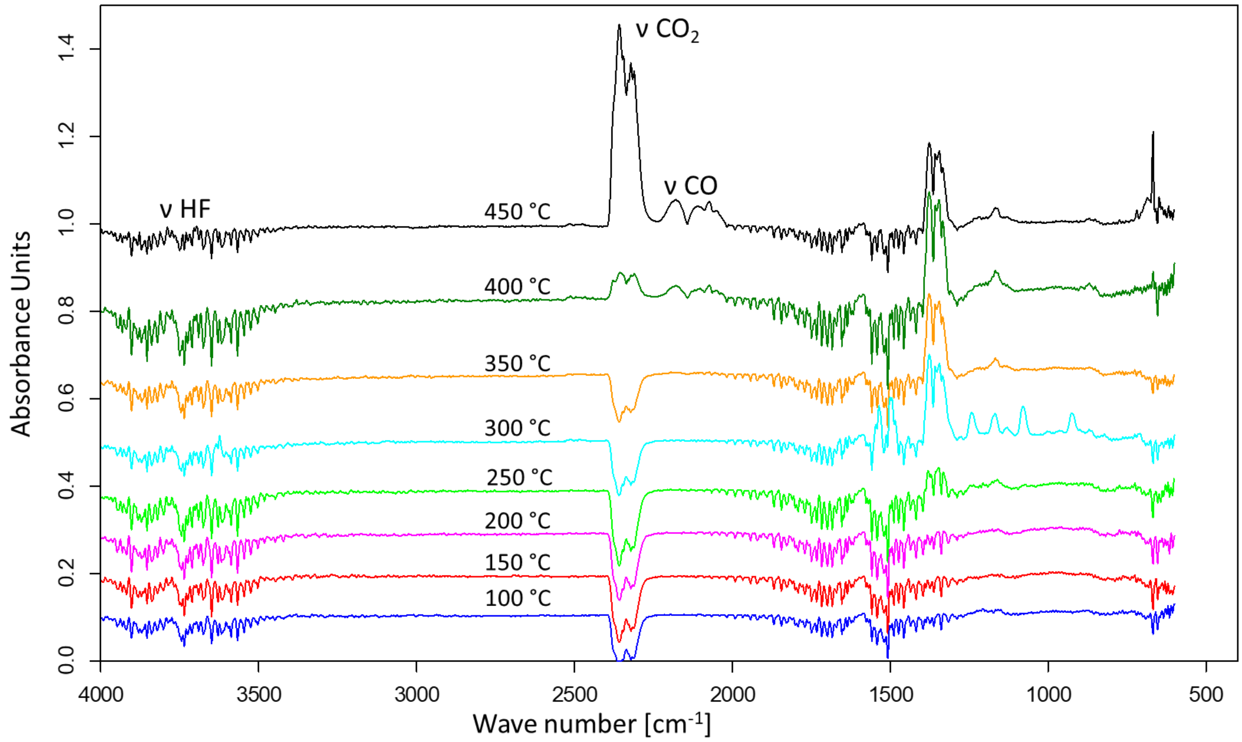


*Figure S10 – FTIR spectra of the gas outlet streams from the TGA measurement of s-PSUs.*

## TGA traces unmodified and modified PSUs

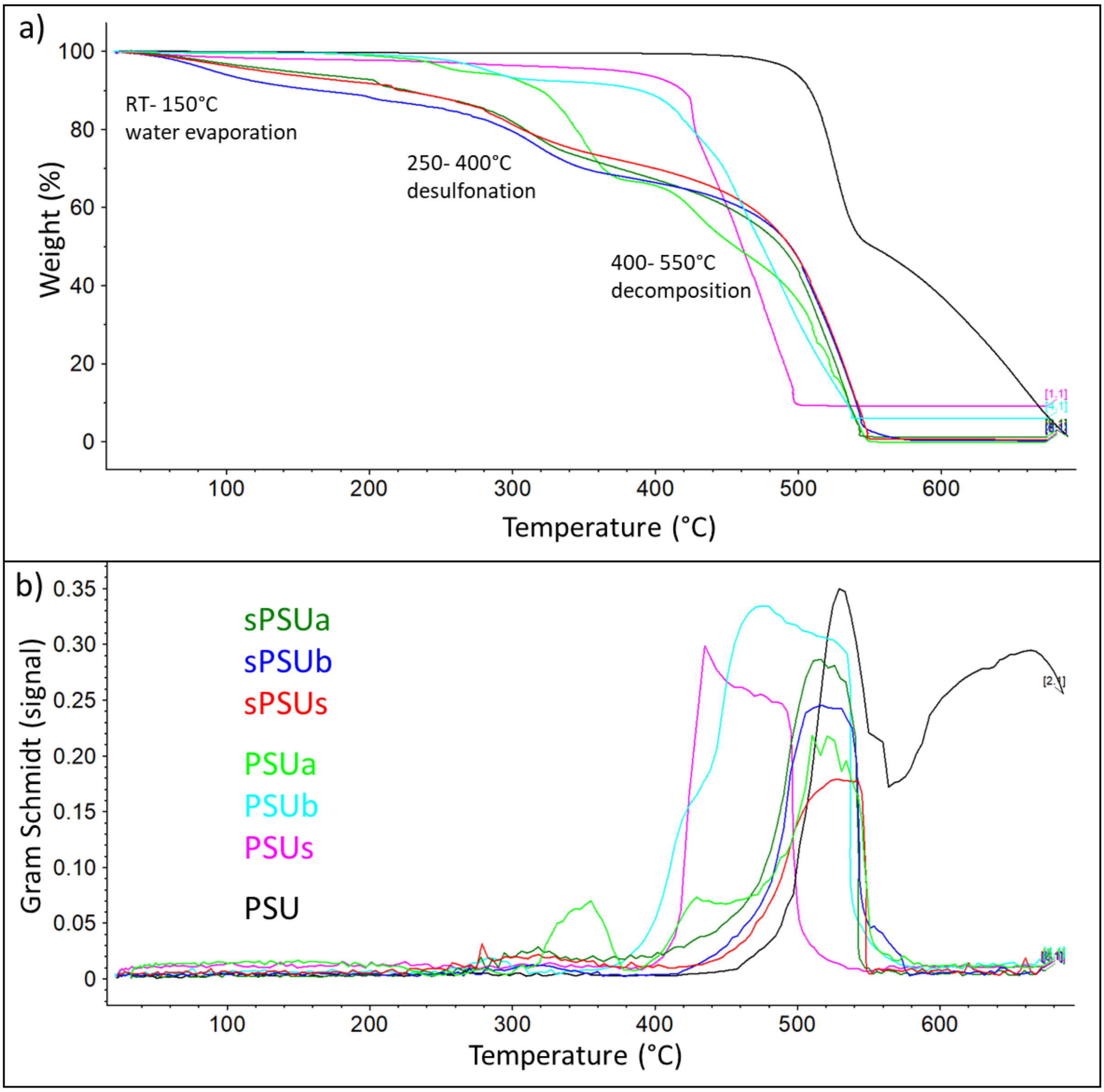


*Figure S11 – TGA curves (a)) and the corresponding Gram Schmidt plot (b)) of s-PSUa, s-PSUb, s-PSUs, and PSU.*

## DSC measurements

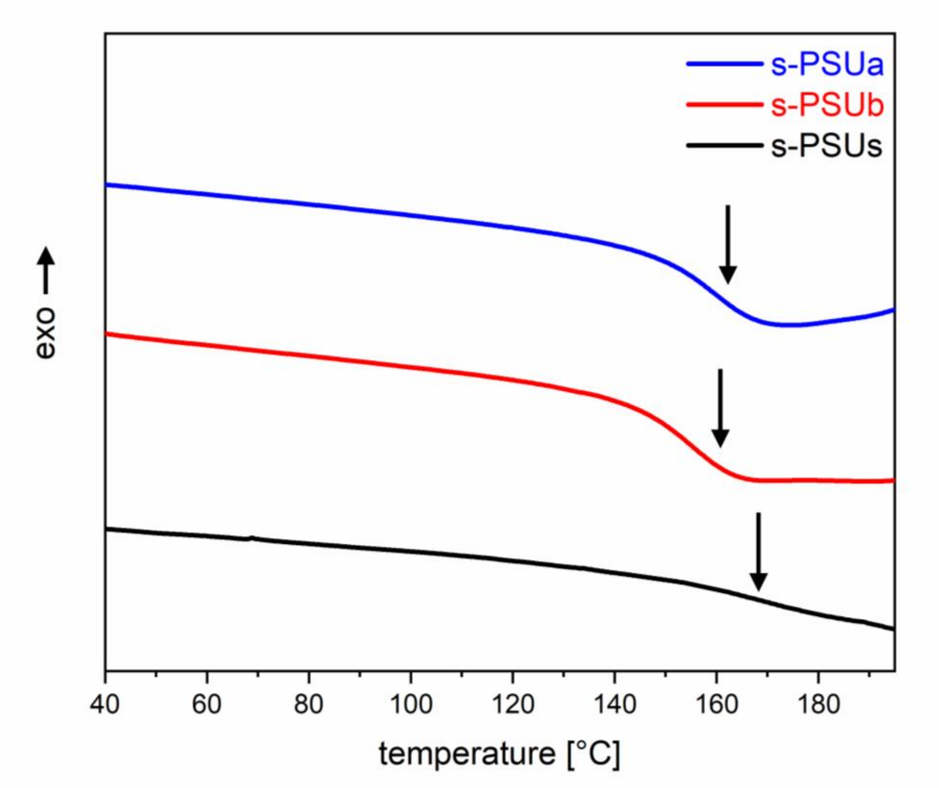


*Figure S12 – DSC-curves (2nd heating cycle) of s-PSUa, s-PSUb and s-PSUs.*

## GPC measurements

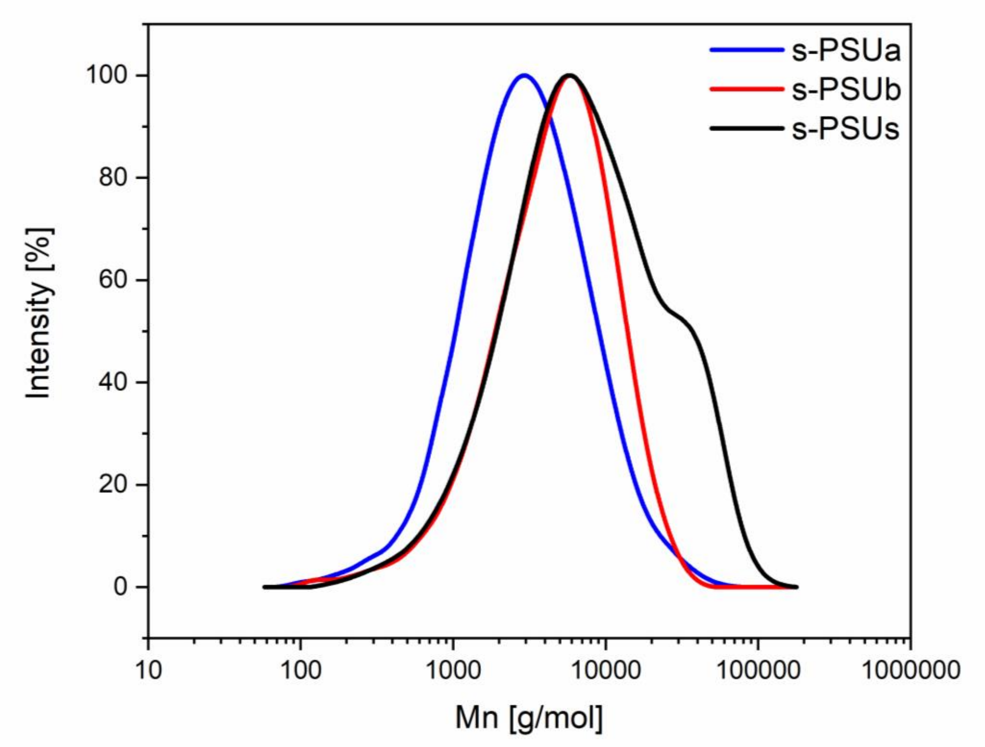


*Figure S13 – GPC-curves of s-PSUa, s-PSUb, and s-PSUs, measured in NMP against polystyrene standards.*

## Reaction scheme s-PSUb

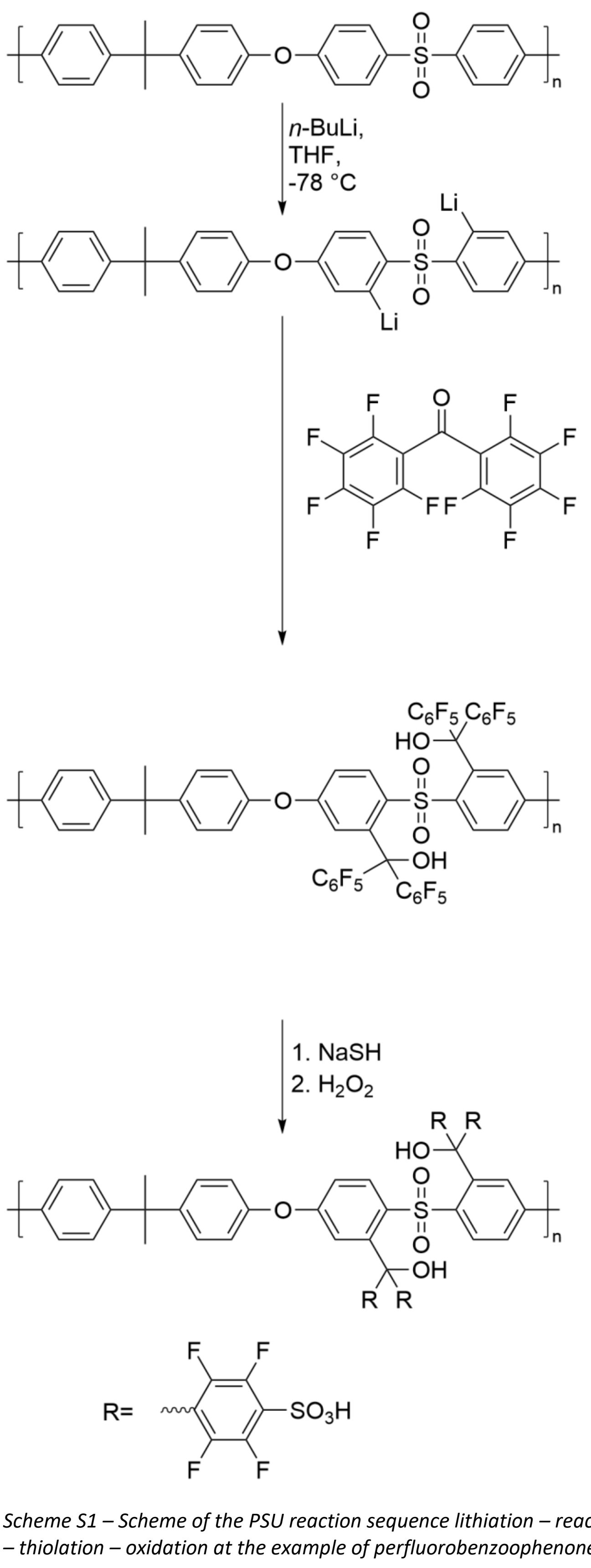


*Scheme S1 – Scheme of the PSU reaction sequence lithiation – reaction with a carbonyl compound (perfluorobenzophenone) – thiolation – oxidation at the example of perfluorobenzoophenone.*

## Reaction scheme s-PSUs

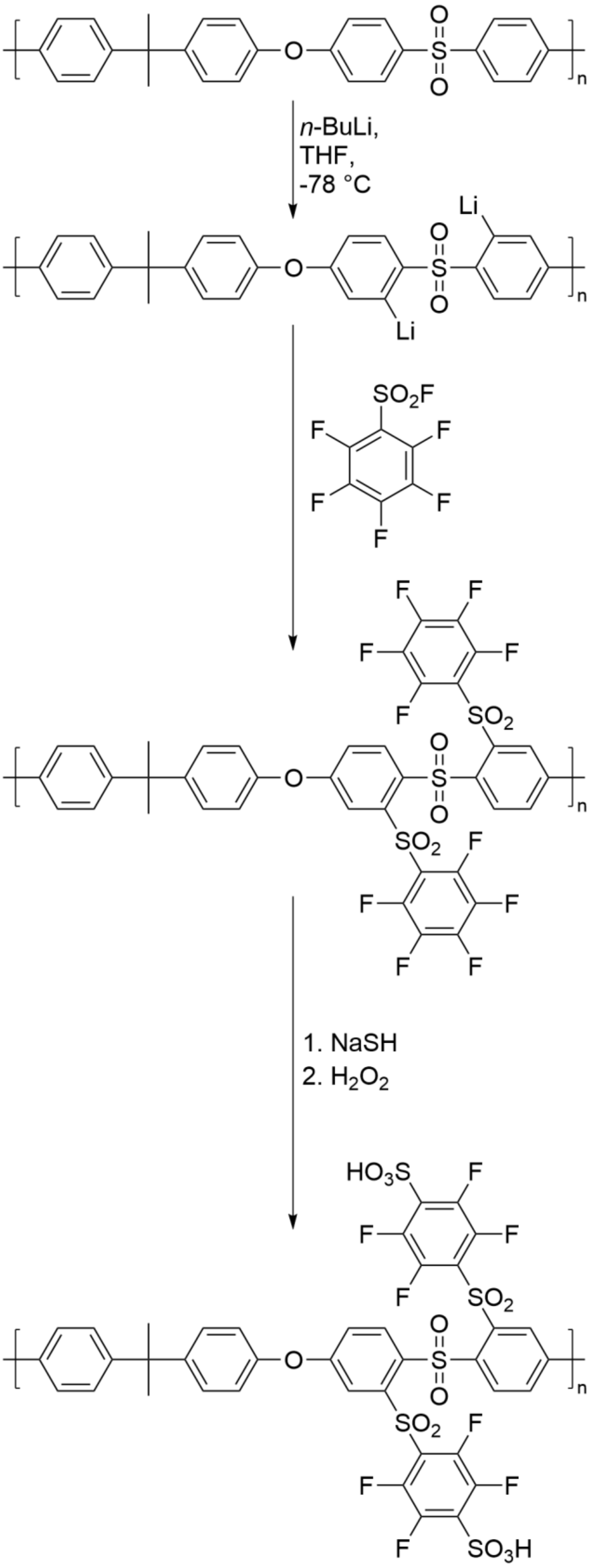


*Scheme S2 - Scheme of the PSU reaction sequence lithiation – reaction with a carbonyl compound (pentafluorobenzenesulfonylfluoride) – thiolation – oxidation at the example of pentafluorobenzenesulfonylfluoride.*

## In-plane swelling data of the membranes

*Table S1 – In-plane swelling data of s-PSUa, s-PSUs, and the blend membrane of OPBI and s-PSUb at room temperature and 85 °C.*

| Sample | $\Delta l_{x-direction,RT}$ $in\ mm$ | $\Delta l_{y-direction,RT}$ $in\ mm$ | $\Delta l_{x-direction,RT}$ $in\ \%$ | $\Delta l_{y-direction,RT}$ $in\ \%$ | $\Delta l_{x-direction,85\ °C}$ $in\ mm$ | $\Delta l_{y-direction,85\ °C}$ $in\ mm$ | $\Delta l_{x-direction,85\ °C}$ $in\ \%$ | $\Delta l_{x-direction,85\ °C}$ $in\ \%$ |
|---|---|---|---|---|---|---|---|---|
| s-PSUa | 1.0 | 1.0 | 6.3 | 8.3 | 4.0 | 2.0 | 19.0 | 15.4 |
| s-PSUs | 1.0 | 1.0 | 4.5 | 5.6 | 7.0 | 8.0 | 58.3 | 88.9 |
| s-PSUb/OPBI (IEC=0,8) | 1.0 | 0.0 | 5.6 | 0.0 | 0.0 | 0.0 | 0.0 | 0.0 |

## Comparison of membrane conductivity to similar works

We focused on PSU-based or similar materials to compare several membrane types to each other. Furthermore, we limited the comparison to membranes characterized by the same measuring cell under similar conditions to show comparable values.

*Table S2: Comparison of membrane conductivity to similar works.*

| Membrane | Polymer/membrane type | Composition | IEC in mmol $g^{-1}$ | σ in mS $cm^{-1}$ |
|---|---|---|---|---|
| 1 | Pentafluorophenyl side-chain modified PSU | s-PSUa[(a)] | 1.15 | 100 |
| 2 | Pentafluorophenyl side-chain modified PSU | s-PSUs[(a)] | 1.09 | 52 |
| 3 | Pentafluorophenyl side-chain modified PSU | s-PSUb/OPBI[(b)] | 0.8 | 55 |
| 4[1] | Acid-base blend membranes with sPEEK/sPSU | 90 wt% sPEEK/ 10 wt% PBI[(b)] | 1.26 | 34.8 |
| 5[1] | Acid-base blend membranes with sPEEK/sPSU | 95 wt% sPSU/ 5 wt% PBI[(b)] | 1.19 | 37.4 |
| 6[1] | Acid-base blend membranes with sPEEK/sPSU | 95 wt% sPEEK/ 5 wt% P4VP | 1.48 | 26.3 |
| 7[1] | Acid-base blend membranes with sPEEK/sPSU | 95 wt% sPSU/ 5 wt% P4VP | 1.36 | 38.7 |
| 8[3] | Covalent/ionically crosslinked blend membranes with sPEEK/sPEK | 54.7 wt% PEEK($SO_3H$)/ 45.3 wt% PSU($SO_2Li$)[(c)] | 1.1 | 43.3 |
| 9[4] | Covalent/ionically crosslinked blend membranes with sPEEK/sPEK/sPSU | 71.6 wt% sulfonated PEK/ 28.4 wt% sulfinated PSU Radel R[(c)] | 1.18 | 102 |
| 10[4] | Covalent/ionically crosslinked blend membranes with sPEEK/sPEK/sPSU | 44.7 wt% sulfonated PEKEKK/55.3 wt% sulfonated PSU Udel[(c)] | 1.2 | 71.2 |
| 11[5] | Ionically crosslinked blend membranes with sulfonated polyarylethers | 2b/PBI[(d)] | 1.12 | 87.7 |
| 12[5] | Ionically crosslinked blend membranes with sulfonated polyarylethers | 3b/PBI[(d)] | 1.21 | 120.5 |
| 13[6] | Covalent/ionically crosslinked blend membranes with s-PEEK/basic modified PSU | PEEKSO2Li-1/basic PSU 2[(e)] | 1.35 | 79.4 |
| 14[6] | Covalent/ionically crosslinked blend membranes with s-PEEK/basic modified PSU | PEEKSO2Li-1/basic PSU 6[(e)] | 1.2 | 70.9 |
| 15[7] | Acid-base blend membranes with partially fluorinated polyethersulfone-copolymers | s-BHPHFP$_{1}$-*co*-s-BHPS$_{0}$/PBI[(b)] | 1.34 | 94.9 |
| 16[7] | Acid-base blend membranes with partially fluorinated polyethersulfone-copolymers | s-BHPHFP$_{0.75}$-*co*-s-BHPS$_{0.25}$/PBI[(b)] | 1.14 | 126.5 |
| 17[7] | Acid-base blend membranes with partially fluorinated polyethersulfone-copolymers | s-BHPHFP$_{0.5}$-*co*-s-BHPS$_{0.5}$/PBI[(b)] | 1.33 | 90.0 |

| | | | | |
|---|---|---|---|---|
| 18[7] | Acid-base blend membranes with partially fluorinated polyethersulfone-copolymers | s-BHPHFP$_{0.25}$-*co*-s-BHPS$_{0.75}$/PBI[(b)] | 1.25 | 146.4 |
| 19[8] | Acid-base blend membranes with poly arylene thioethers/PBI | SPTS40/PBI[(b)] | 1.15 | 150 |
| 20[8] | Acid-base blend membranes with poly arylene thioethers/PBI | SPTS50/PBI[(b)] | 1.17 | 250 |
| 21[8] | Acid-base blend membranes with poly arylene thioethers/PBI | SPTES50/PBI[(b)] | 1.11 | 120 |
| 22[8] | Acid-base blend membranes with poly arylene thioethers/PBI | SPTEK50/PBI[(b)] | 1.10 | 220 |
| 23[9] | Sulfonated multiblock-co-ionomers based on poly aryl(thio)ethers | 1b[(a)] | 1.13 | 248 |
| 24[9] | Sulfonated multiblock-co-ionomers based on poly aryl(thio)ethers | 2b[(a)] | 0.92 | 239 |
| 25[9] | Sulfonated multiblock-co-ionomers based on poly aryl(thio)ethers | 3b[(a)] | 1.07 | 280 |
| 26[9] | Sulfonated multiblock-co-ionomers based on poly aryl(thio)ethers | 4b[(a)] | 0.87 | 190 |
| 27[10] | Sulfonated partially fluorinated polyarylene ethers | 3b[(a)] | 1.19 | 99 |
| 28[10] | Sulfonated partially fluorinated polyarylene ethers | 4b[(a)] | 0.83 | 36 |
| 29[10] | Sulfonated partially fluorinated polyarylene ethers | 4c[(a)] | 1.48 | 167 |
| 30[11] | Blend membranes with poly(arylene sulfone)/PBI | SFS-PBIOO[(b)] | 0.8 | 42 |
| 31[11] | Blend membranes with poly(arylene sulfone)/PBI | SPSU-PBIOO[(b)] | 0.7 | 24 |
| 32[11] | Blend membranes with poly(arylene sulfone)/PBI | SPEEK-PBIOO[(b)] | 0.7 | 27 |
| 33[11] | Blend membranes with poly(arylene sulfone)/PBI | SPSO-PBIOO[(b)] | 0.7 | 110 |
| 34[12] | Acid-base blend membranes with sulfonated multiblock copolymers/aryl polymers | MBI-LS[(a)] | 0.98 | 75.2 |
| 35[12] | Acid-base blend membranes with sulfonated multiblock copolymers/aryl polymers | MBI-LL[(a)] | 0.52 | 58.5 |
| 36[12] | Acid-base blend membranes with sulfonated multiblock copolymers/aryl polymers | MBI-MS[(a)] | 1.1 | 102 |
| 37[12] | Acid-base blend membranes with sulfonated multiblock copolymers/aryl polymers | SFS 85 wt%/PSU-py 12 wt%/F6PBI 3 wt%[(e)] | 1.38 | 56.3 |
| 38[12] | Acid-base blend membranes with sulfonated multiblock copolymers/aryl polymers | SFS 85 wt%/PSU-py 10 wt%/F6PBI 5 wt%[(e)] | 1.19 | 137.7 |

[(a)]Pure membrane, [(b)]acid-base blend membrane, [(c)]covalent crosslinked membrane, [(d)]ionic crosslinked membrane, [(e)]ternary acid-base blend-membrane

## References

(1) Kerres, J.; Ullrich, A.; Haering, T.; Baldauf, M.; Gebhardt, U.; Preidel, W. Preparation, characterization, and fuel cell application of new acid-base blend membranes. *Journal of New Materials for Electrochemical Systems* **2000**, *3* (3), 229–239.
(2) Guiver, M. D.; Apsimon, J. W.; Kutowy, O. The modification of polysulfone by metalation. *J. Polym. Sci. B Polym. Lett. Ed.* **1988**, *26* (2), 123–127. DOI: 10.1002/pol.1988.140260211.
(3) Kerres, J.; Zhang, W.; Jorissen, L.; Gogel, V. Application of different types of polyaryl-blend-membranes in DMFC. *Journal of New Materials for Electrochemical Systems* **2002**, *5*, 97–107.
(4) Kerres, J.; Zhang, W.; Ullrich, A.; Tang, C.-M.; Hein, M.; Gogel, V.; Frey, T.; Jörissen, L. Synthesis and characterization of polyaryl blend membranes having different composition, different covalent and/or ionical crosslinking density, and their application to DMFC. *Desalination* **2002**, *147* (1-3), 173–178. DOI: 10.1016/S0011-9164(02)00530-1.
(5) Kerres, J. A.; Xing, D.; Schönberger, F. Comparative investigation of novel PBI blend ionomer membranes from nonfluorinated and partially fluorinated poly arylene ethers. *J. Polym. Sci. B Polym. Phys.* **2006**, *44* (16), 2311–2326. DOI: 10.1002/polb.20862.
(6) Kerres, J. Covalent-Ionically Cross-linked Poly(Etheretherketone)-Basic Polysulfone Blend Ionomer Membranes. *Fuel Cells* **2006**, *6* (3-4), 251–260. DOI: 10.1002/fuce.200500207.
(7) SCHONBERGER, F.; Hein, M.; Kerres, J. Preparation and characterisation of sulfonated partially fluorinated statistical poly(arylene ether sulfone)s and their blends with PBI. *Solid State Ionics* **2007**, *178* (7-10), 547–554. DOI: 10.1016/j.ssi.2007.01.003.
(8) Lee, J. K.; Kerres, J. Synthesis and characterization of sulfonated poly(arylene thioether)s and their blends with polybenzimidazole for proton exchange membranes. *Journal of Membrane Science* **2007**, *294* (1-2), 75–83. DOI: 10.1016/j.memsci.2007.02.013.
(9) Schönberger, F.; Kerres, J. Novel multiblock-co-ionomers as potential polymer electrolyte membrane materials. *J. Polym. Sci. A Polym. Chem.* **2007**, *45* (22), 5237–5255. DOI: 10.1002/pola.22269.
(10) Schönberger, F.; Chromik, A.; Kerres, J. Synthesis and characterization of novel (sulfonated) poly(arylene ether)s with pendent trifluoromethyl groups. *Polymer* **2009**, *50* (9), 2010–2024. DOI: 10.1016/j.polymer.2009.02.043.
(11) Katzfuß, A.; Krajinovic, K.; Chromik, A.; Kerres, J. Partially fluorinated sulfonated poly(arylene sulfone)s blended with polybenzimidazole. *J. Polym. Sci. A Polym. Chem.* **2011**, *49* (8), 1919–1927. DOI: 10.1002/pola.24624.
(12) Bender, J.; Mayerhöfer, B.; Trinke, P.; Bensmann, B.; Hanke-Rauschenbach, R.; Krajinovic, K.; Thiele, S.; Kerres, J. H+-Conducting Aromatic Multiblock Copolymer and Blend Membranes and Their Application in PEM Electrolysis. *Polymers* **2021**, *13* (20). DOI: 10.3390/polym13203467. Published Online: Oct. 9, 2021.
